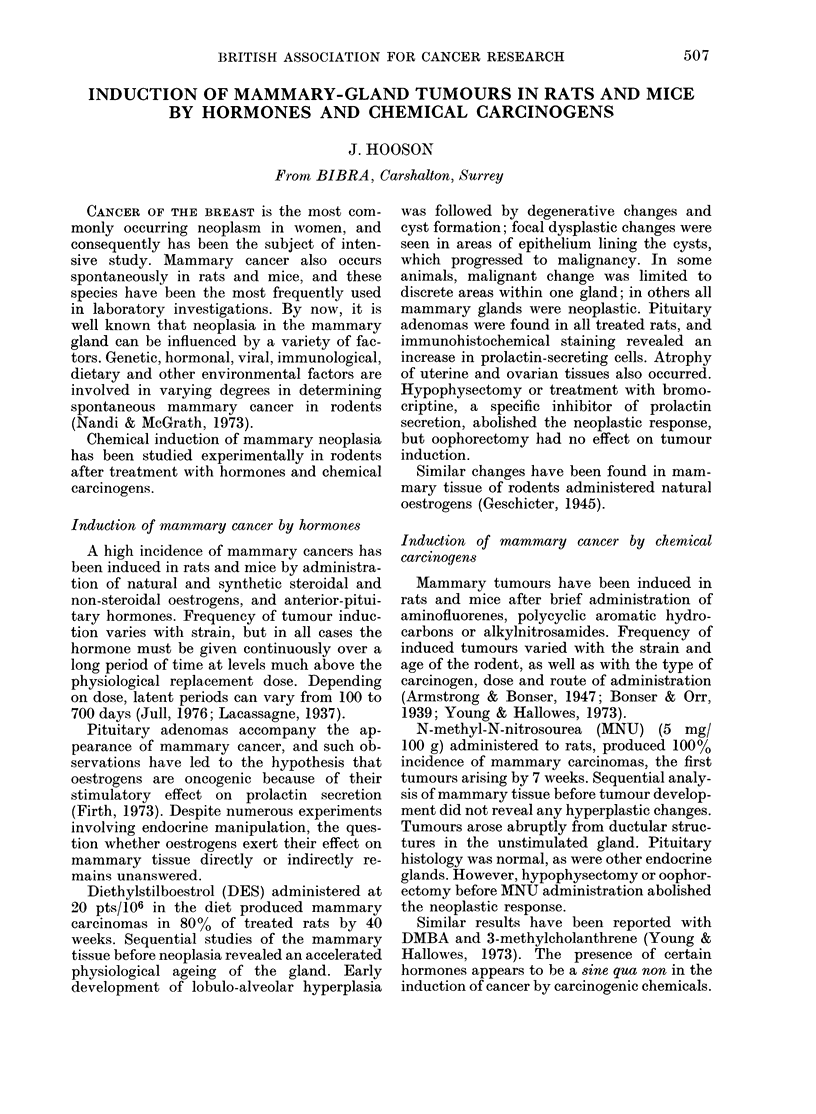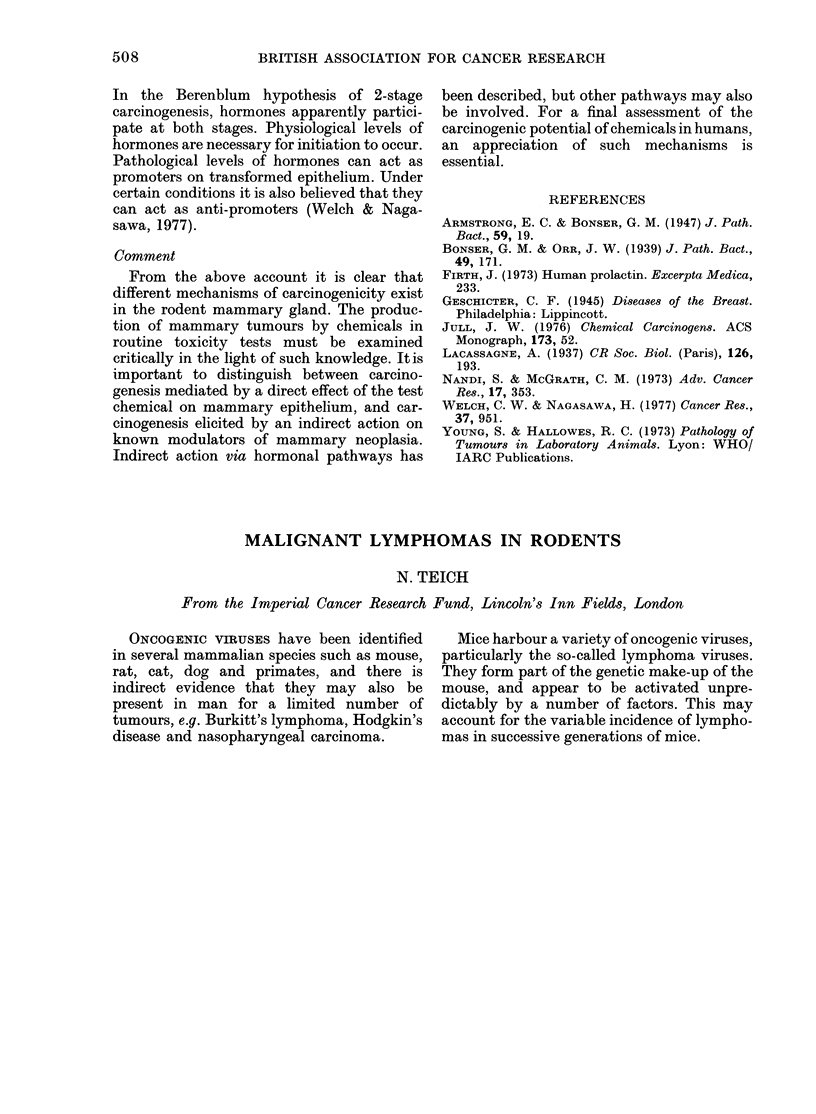# Induction of mammary-gland tumours in rats and mice by hormones and chemical carcinogens.

**DOI:** 10.1038/bjc.1980.86

**Published:** 1980-03

**Authors:** J. Hooson


### INDUCTION OF MAMMARY-GLAND TUMOURS IN RATS AND MICE BY HORMONES AND CHEMICAL CARCINOGENS

J. HOOSON

*From BIBRA, Carshalton, Surrey*

CANCER OF THE BREAST is the most commonly occurring neoplasm in women, and consequently has been the subject of intensive study. Mammary cancer also occurs spontaneously in rats and mice, and these species have been the most frequently used in laboratory investigations. By now, it is well known that neoplasia in the mammary gland can be influenced by a variety of factors. Genetic, hormonal, viral, immunological, dietary and other environmental factors are involved in varying degrees in determining spontaneous mammary cancer in rodents (Nandi & McGrath, 1973).

Chemical induction of mammary neoplasia has been studied experimentally in rodents after treatment with hormones and chemical carcinogens.

### *Induction of mammary cancer by hormones*

A high incidence of mammary cancers has been induced in rats and mice by administration of natural and synthetic steroidal and non-steroidal oestrogens, and anterior-pituitary hormones. Frequency of tumour induction varies with strain, but in all cases the hormone must be given continuously over a long period of time at levels much above the physiological replacement dose. Depending on dose, latent periods can vary from 100 to 700 days (Jull, 1976; Lacassagne, 1937).

Pituitary adenomas accompany the appearance of mammary cancer, and such observations have led to the hypothesis that oestrogens are oncogenic because of their stimulatory effect on prolactin secretion (Firth, 1973). Despite numerous experiments involving endocrine manipulation, the question whether oestrogens exert their effect on mammary tissue directly or indirectly remains unanswered.

Diethylstilboestrol (DES) administered at 20 pts/$10^6$ in the diet produced mammary carcinomas in 80% of treated rats by 40 weeks. Sequential studies of the mammary tissue before neoplasia revealed an accelerated physiological ageing of the gland. Early development of lobulo-alveolar hyperplasia was followed by degenerative changes and cyst formation; focal dysplastic changes were seen in areas of epithelium lining the cysts, which progressed to malignancy. In some animals, malignant change was limited to discrete areas within one gland; in others all mammary glands were neoplastic. Pituitary adenomas were found in all treated rats, and immunohistochemical staining revealed an increase in prolactin-secreting cells. Atrophy of uterine and ovarian tissues also occurred. Hypophysectomy or treatment with bromocriptine, a specific inhibitor of prolactin secretion, abolished the neoplastic response, but oophorectomy had no effect on tumour induction.

Similar changes have been found in mammary tissue of rodents administered natural oestrogens (Geschicter, 1945).

### *Induction of mammary cancer by chemical carcinogens*

Mammary tumours have been induced in rats and mice after brief administration of aminofluorenes, polycyclic aromatic hydrocarbons or alkylnitrosamides. Frequency of induced tumours varied with the strain and age of the rodent, as well as with the type of carcinogen, dose and route of administration (Armstrong & Bonser, 1947; Bonser & Orr, 1939; Young & Hallowes, 1973).

N-methyl-N-nitrosourea (MNU) (5 mg/100 g) administered to rats, produced 100% incidence of mammary carcinomas, the first tumours arising by 7 weeks. Sequential analysis of mammary tissue before tumour development did not reveal any hyperplastic changes. Tumours arose abruptly from ductular structures in the unstimulated gland. Pituitary histology was normal, as were other endocrine glands. However, hypophysectomy or oophorectomy before MNU administration abolished the neoplastic response.

Similar results have been reported with DMBA and 3-methylcholanthrene (Young & Hallowes, 1973). The presence of certain hormones appears to be a *sine qua non* in the induction of cancer by carcinogenic chemicals.

In the Berenblum hypothesis of 2-stage carcinogenesis, hormones apparently participate at both stages. Physiological levels of hormones are necessary for initiation to occur. Pathological levels of hormones can act as promoters on transformed epithelium. Under certain conditions it is also believed that they can act as anti-promoters (Welch & Nagasawa, 1977).

*Comment*

From the above account it is clear that different mechanisms of carcinogenicity exist in the rodent mammary gland. The production of mammary tumours by chemicals in routine toxicity tests must be examined critically in the light of such knowledge. It is important to distinguish between carcinogenesis mediated by a direct effect of the test chemical on mammary epithelium, and carcinogenesis elicited by an indirect action on known modulators of mammary neoplasia. Indirect action *via* hormonal pathways has been described, but other pathways may also be involved. For a final assessment of the carcinogenic potential of chemicals in humans, an appreciation of such mechanisms is essential.

REFERENCES

ARMSTRONG, E. C. & BONSER, G. M. (1947) *J. Path. Bact.*, **59**, 19.

BONSER, G. M. & ORR, J. W. (1939) *J. Path. Bact.*, **49**, 171.

FIRTH, J. (1973) Human prolactin. *Excerpta Medica*, 233.

GESCHICTER, C. F. (1945) *Diseases of the Breast.* Philadelphia: Lippincott.

JULL, J. W. (1976) *Chemical Carcinogens.* ACS Monograph, **173**, 52.

LACASSAGNE, A. (1937) *CR Soc. Biol.* (Paris), **126**, 193.

NANDI, S. & MCGRATH, C. M. (1973) *Adv. Cancer Res.*, **17**, 353.

WELCH, C. W. & NAGASAWA, H. (1977) *Cancer Res.*, **37**, 951.

YOUNG, S. & HALLOWES, R. C. (1973) *Pathology of Tumours in Laboratory Animals.* Lyon: WHO/IARC Publications.